# Evidence for Cryptic Speciation in Directly Transmitted Gyrodactylid Parasites of Trinidadian Guppies

**DOI:** 10.1371/journal.pone.0117096

**Published:** 2015-01-09

**Authors:** Raquel Xavier, Patricia J. Faria, Giuseppe Paladini, Cock van Oosterhout, Mireille Johnson, Jo Cable

**Affiliations:** 1 School of Biological Sciences, Cardiff University, Cardiff, CF10 3AX, United Kingdom; 2 CIBIO, Centro de Investigação em Biodiversidade e Recursos Genéticos, Universidade do Porto, Campus Agrário de Vairão, 4485–661 Vairão, Portugal; 3 Institute of Aquaculture, School of Natural Sciences, University of Stirling, Stirling, FK9 4LA, United Kingdom; 4 School of Environmental Sciences, University of East Anglia, Norwich Research Park, Norwich NR4 7TJ, United Kingdom; Emory University, United States of America

## Abstract

Cryptic species complexes are common among parasites, which tend to have large populations and are subject to rapid evolution. Such complexes may arise through host-parasite co-evolution and/or host switching. For parasites that reproduce directly on their host, there might be increased opportunities for sympatric speciation, either by exploiting different hosts or different micro-habitats within the same host. The genus *Gyrodactylus* is a specious group of viviparous monogeneans. These ectoparasites transfer between teleosts during social contact and cause significant host mortality. Their impact on the guppy (*Poecilia reticulata*), an iconic evolutionary and ecological model species, is well established and yet the population genetics and phylogenetics of these parasites remains understudied. Using mtDNA sequencing of the host and its parasites, we provide evidence of cryptic speciation in *Gyrodactylus bullatarudis*, *G. poeciliae* and *G. turnbulli*. For the COII gene, genetic divergence of lineages within each parasite species ranged between 5.7 and 17.2%, which is typical of the divergence observed between described species in this genus. Different lineages of *G. turnbulli* and *G. poeciliae* appear geographically isolated, which could imply allopatric speciation. In addition, for *G. poeciliae*, co-evolution with a different host species cannot be discarded due to its host range. This parasite was originally described on *P. caucana*, but for the first time here it is also recorded on the guppy. The two cryptic lineages of *G. bullatarudis* showed considerable geographic overlap. *G. bullatarudis* has a known wide host range and it can also utilize a killifish (*Anablepsoides hartii*) as a temporary host. This killifish is capable of migrating overland and it could act as a transmission vector between otherwise isolated populations. Additional genetic markers are needed to confirm the presence of these cryptic *Gyrodactylus* species complexes, potentially leading to more in-depth genetic, ecological and evolutionary analyses on this multi-host-parasite system.

## Introduction

Comparative phylogeographic studies have demonstrated co-evolution between parasites and their hosts [1, 2], yet co-speciation does not appear to be a major factor in host-parasite evolution [3]. Many confounding factors such as multi-host systems, extinction and host-switching can cause incongruence between host and parasite phylogenies [4, 5]. Parasites typically evolve faster than their hosts [6–8] related to their shorter generation times that may result in higher substitution rates [9]. Although hosts and parasites may be involved in a co-evolutionary arms race in which adaptive evolution is driven by natural selection, parasite transmission often results in serial population bottlenecks and founder events, which can have a significant role in driving parasite evolution [6–8]. Such demographic dynamics will leave a distinct phylogenetic and population genetic signature, possibly leading to an increase in the genetic differentiation of parapatric/allopatric parasite populations, which can ultimately result in insipient speciation [10].

Monogeneans are particularly suitable parasites for revealing novel insights into host ecology and evolution [11, 12], due to their direct life-cycles and relatively high level of host-preference. Species from the genus *Gyrodactylus* belong to the most studied group of monogeneans, renowned for their impact on aquaculture and conservation planning. They parasitize many wild and farmed fish, including Atlantic salmon (*Salmo salar*) and brown trout (*Salmo trutta*) [13], and because they reproduce *in situ* on the host and are transmitted during host contact, epidemics can sweep quickly through fish populations. Although more than 400 species have been described for the genus, *Gyrodactylus* species are morphologically conserved and this is probably related to the adaptations associated with viviparity and progenesis [14, 15]. For this reason, molecular data is often essential for confirmation of species descriptions [16, 17] and discrimination [15–19]. Genetic studies have uncovered otherwise cryptic species [20] and demonstrated that *Gyrodactylus* diversity may be underestimated [17]. This is likely to be the case also for the well-studied guppy-gyrodactylid system in Trinidad [21].

*Poecilia reticulata* (guppy)—*Gyrodactylus bullatarudis*/*G. turnbulli* associations from Trinidadian rivers have been extensively examined in order to understand how parasitism can impact a host, namely affecting body size [22], reproduction [23–25], immune response and survival [26–28]. Trinidadian guppy-gyrodactylid dyads have also been studied to evaluate parasite transmission dynamics in social hosts [29, 30] and to test the impacts of infections on conservation strategies involving reintroduction from captive-breeding programs [31, 32]. Although the two parasite species can infect the same host species, they are very distinct phylogenetically [15, 33]. *G. bullatarudis* can also use a broader host range than *G. turnbulli*, both under laboratory [34, 35] and field [36] conditions. Relatively little is known about the biology of other poeciliid gyrodactylids, including *G. poeciliae*, which is closely related to *G. bullatarudis* [15]. Despite the obvious importance of this group of parasites to the evolutionary ecology of their hosts, very little is known about their molecular ecology. In fact the only record present in GenBank for *G. bullatarudis*, *G. poeciliae* and *G. turnbulli* (four sequences in total) were generated to confirm specific status and address phylogenetic questions [15, 21, 37].

In contrast, the genetic diversity and differentiation of guppy populations from Northern Trinidad has been the focus of many studies over the last three decades; based on this knowledge we can make specific predictions about the genetic diversity of their parasite species. Originally, based on allozyme and mitochondrial data, Shaw *et al*. [38] and Fajen and Breden [39] argued that guppies from Northern Trinidad form at least two genetically distinct groups, possibly originating from two different colonisation events of the Northern-Caroni and Oropouche drainages, and from different ancestral stocks. Recent studies, however, using microsatellites and SNPs indicate that colonisation of Trinidadian rivers was much more complex [40, 41]. In general, all studies are in agreement in detecting high levels of genetic differentiation between populations from different drainages due to genetic drift and adaptive selection [41]. Population differentiation has also been detected between upland and lowland sites in the Marianne River (Northern drainage) and rivers from the Caroni drainage, related to genetic drift caused by geographic distance and physical barriers such as waterfalls [42, 43]. Guppy migration within drainages mainly occurs in a downstream direction with headwater populations generally being more isolated and less genetically diverse [42, 43]. Migration does occur between rivers, but typically in lowland stretches during wet season flooding, as demonstrated by high levels of genetic diversity of lowland populations [38, 43].

We hypothesized that, due to the high level of phylogeographic structure previously reported amongst host populations and the high levels of host-specificity reported for *Gyrodactylus* species, parasite speciation might have led to multiple cryptic species. Furthermore, as *G. bullatarudis* uses alternative hosts and has a higher potential for host switching and migration, it is likely to show a lower level of genetic differentiation between rivers when compared to more host-specific *Gyrodactylus* species. Thus, the present study aimed to examine the genetic diversity and phylogeographic patterns of *Gyrodactylus* species infecting guppies (*P. reticulata*) from five rivers of North Trinidad using partial sequences of the mitochondrial cytochrome oxidase II (COII) gene. In addition, the phylogeographic structure of guppies was evaluated through sequences of a portion of the first domain (HVR) of mitochondrial control region (D-loop) to compare with that of the parasites and to test the levels of host-specificity. Our results seem to confirm both our hypotheses and provide the first evidence that cryptic speciation might have occurred within the three *Gyrodactylus* spp. studied.

## Material and Methods

### Ethics Statement

This work was conducted using the guppy (*Poecilia reticulata*)–*Gyrodactylus turnbulli*/ *G. bullatarudis*/ *G. poeciliae* model systems. All fish were collected, handled and killed [44] according to UK Home Office Project license (PPL 30/2357) regulations and approved by the Cardiff University Ethics Committee. In Trinidad there is no legislation restricting collection of fishes from public areas (and none of our sites were located or accessed via privately owned land) and *P. reticulata* is not an endangered or protected species. Map reference coordinates for sampled sites are as follows: Lower Aripo 694410E, 1177783N; Upper Aripo 694030E, 1182128N; Lower Lopinot 683553E, 1175663N; Upper Lopinot 683520E, 1182443N; Lower Marianne 685890E, 1193642N; Upper Marianne 685891E, 1192747N; Lower Oropouche 0704394E, 1178967N; Upper Oropouche 0700467E, 1183194N; and Upper Yarra 683427E, 1189518N.

### Fish collection and morphological identification of *Gyrodactylus* spp.

Between October 2003 and November 2004, 230 specimens of *P. reticulata* were collected from 9 different locations of the Caroni (Lower and Upper Aripo River and Lower and Upper Lopinot River), Northern (Upper Yarra, Upper and Lower Marianne River) and the Oropouche drainages (Upper and Lower Oropouche), Trinidad, and stored individually in eppendorfs pre-filled with 90% ethanol for subsequent molecular analysis (see Table 1 for details, and Fig. 1 in Willing *et al*. [41] for drainages and Fig. 1 in Barson *et al*. [43] for details of Caroni rivers). The samples were transported back to Cardiff and specimens of *Gyrodactylus* were collected from the fins and body of guppies, individually placed in a drop of water on a glass slide and bisected in half using fine needles under a dissecting-microscope. The posterior half of the worm was partially digested using the method detailed by Paladini *et al*. [45], and the specimens initially identified according to Harris & Cable [21] and Cable *et al*. [37]. A total of 131 *Gyrodactylus* were identified to species level in this way and the anterior half of each parasite was kept for molecular analysis in 90% ethanol (Table 1). Our initial plan was to obtain rDNA Internal Transcribed Spacers (ITS) and microsatellites data from each individual parasite, but due to difficulties encountered with the mtDNA sequencing, which required multiple PCRs per worm due to high PCR failure rates from individual worms, this was not possible given the limited amount of DNA remaining. For some specimens, DNA was extracted only from the anterior part of the parasite, measuring less than 0.2 mm and containing fewer than 200 cells [15, 46], sufficient in theory for three PCRs. If this failed, DNA was extracted from the remainder of the parasite.

**Table 1:** Number of individuals sampled (N), number of haplotypes (H), Haplotype diversity (Hd) and nucleotide diversity (π) per locality for each lineage within *Gyrodactylus* morphospecies *G. turnbulli* (GtM, GtC and GtO), *G. poeciliae* (GpY and GpCM), and *G. bullatarudis* (Gb1 and Gb2), and for their fish host, *Poecilia reticulata*.

**Morphospecies**	Lineage		**ARIPO**	Upper Aripo	Lower Aripo	**LOPINOT**	Upper Lopinot	Lower Lopinot	**OROPOUCHE**	Upper Oroupoche	Lower Oroupoche	**MARIANNE**	Upper Marianne	Lower Marianne	**UPPER YARRA**	Total
***G. turnbulli***	GtC	***N***	**7**	6	1	**5**	2	3	-	-	-	-	-	-	-	**12**
		**H**	**2**	1	1	**3**	2	1	-	-	-	-	-	-	-	**4**
		**Hd**	**0.29**	0	-	**0.7**	0.5	0	-	-	-	-	-	-	-	**0.731**
		**π**	**0.006**	0	-	**0.011**	0.004	0	-	-	-	-	-	-	-	**0.024**
	GtO	***N***	-	-	-	-	-	-	**1**	-	1	-	-	-	-	**1**
		**H**	-	-	-	-	-	-	**1**	-	1	-	-	-	-	**1**
		**Hd**	-	-	-	-	-	-	-	-	-	-	-	-	-	-
		**π**	-	-	-	-	-	-	-	-	-	-	-	-	-	-
	GtM	***N***	-	-	-	-	-	-	-	-	-	**12**	-	12	-	**12**
		**H**	-	-	-	-	-	-	-	-	-	**2**	-	2	-	**2**
		**Hd**	-	-	-	-	-	-	-	-		**0.409**		0.409	**0.409**	**0.409**
		**π**	-	-	-	-	-	-	-	-	-	**0.000**	-	0.000	-	**0.000**
***G. poeciliae***	**GpCM**	***N***	**12**	3	9	**6**	5	1	-	-	-	**40**	17	25	-	**60**
		**H**	**2**	1	1	**3**	2	1	-	-	-	**3**	1	3	-	**6**
		**Hd**	**0.41**	0	0	**0.6**	0.4	-	-	-	-	**0.5**	0	0.24	-	**0.485**
		**π**	**0.006**	0	0	**0.008**	0.002	-	-	-	-	**0.002**	0	0.003	-	**0.007**
	**GpY**	***N***	-	-	-	-	-	-	-	-	-	-	-	-	**2**	**2**
		**H**	-	-	-	-	-	-	-	-	-	-	-	-	**1**	**1**
		**Hd**	-	-	-	-	-	-	-	-	-	-	-	-	**0**	**0**
		**π**	-	-	-	-	-	-	-	-	-	-	-	-	**0**	**0**
***G. bullatarudis***	**Gb1**	***N***	**16**	5	11	**5**	-	5	**11**	-	11	-	-			**32**
		**H**	**2**	1	2	**1**	-	1	**1**	-	1	-	-	-	-	**2**
		**Hd**	**0.125**	0	0.182	**0**	-	0	**0**	-	0	-	-	-	-	**0.315**
		**π**	**0.001**	0	0.007	**0**	-	0	**0**	-	0	-	-	-	-	**0.001**
	**Gb2**	***N***	**5**	5	-	**1**	-	1	**6**	6	-	-	-	-	-	**12**
		**H**	**1**	1	-	**1**	-	1	**1**	1	-	-	-	-	-	**2**
		**Hd**	**0**	0	-	-	-	-	**0**	0	-	-	-	-	-	**0.167**
		**π**	**0**	0	-	-	-	n.a	**0**	0	-	-	-	-	-	**0.001**
***P. reticulata***		***N***	**61**	36	25	**57**	25	32	**50**	24	26	**62**	27	35	-	**230**
		**H**	**6**	5	3	**7**	3	5	**4**	3	4	**7**	5	5	-	**22**
		**Hd**	**0.60**	0.39	0.35	**0.79**	0.64	0.69	**0.76**	0.68	0.75	**0.60**	0.64	0.55	-	**0.905**
		**π**	**0.003**	0.001	0.004	**0.023**	0.002	0,019	**0.003**	0.003	0.003	**0.004**	0.004	0.003	-	**0.023**

**Figure 1:**
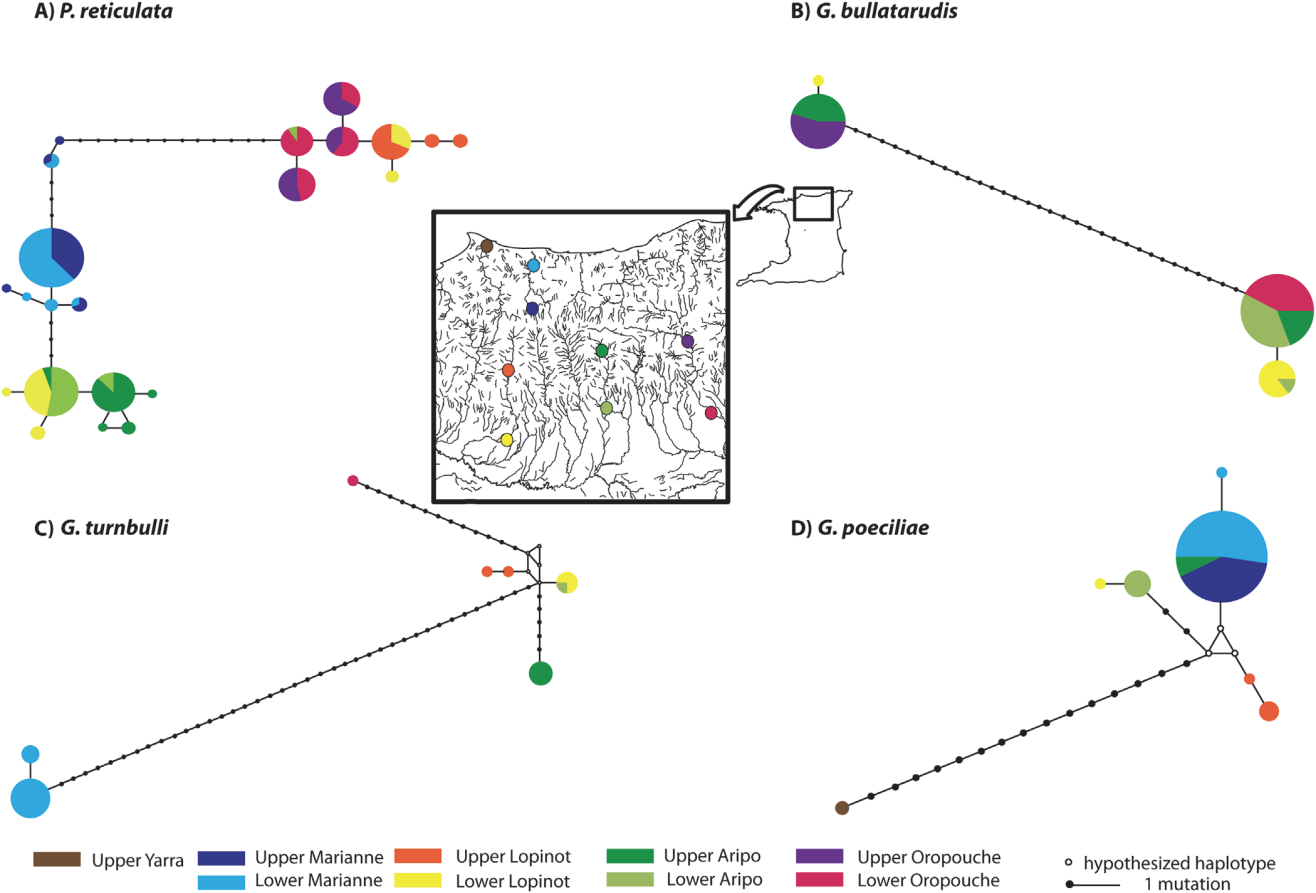
Median joining haplotype networks estimated for (A) *Poecilia reticulata*, and (B-D) for each *Gyrodactylus* species from nine localities in the Northern Mountain Range of Trinidad.

### DNA extraction, PCR amplification and sequencing

For *P. reticulata*, genomic DNA was extracted from the caudal fin using the HotSHOT protocol [47]. A total of 414 bp of the first domain (HVR) of the mitochondrial (mt) D-loop was amplified using primer pairs L15926 and H16498 [48, 49] according to the following PCR conditions: 95°C for 3 minutes and 35 cycles at 95°C for 30 seconds, 54°C for 30 seconds and 72°C for 1 minute. For *Gyrodactylus* spp. genomic DNA was extracted using DNeasy Blood & Tissue QIAGEN kit. A 262 bp fragment of the mt protein coding COII gene was amplified using the QIAGEN Multiplex PCR kit and primer pairs COX2 F1 (TACATAYCGCCCGTCAATYT) and COX2 R1 (TCARTAYCACTGDCGDCCYA), developed specifically for this study, and following PCR protocol of initial denaturation temperature of 95°C for 15 minutes and 40 cycles at 95°C for 30 seconds, 50°C for 90 seconds and 72°C for 90 seconds. All PCR products were cleaned using Exo/AP and sequenced by a commercial company. All sequences were deposited in GenBank (Accession Numbers KP168263-KP168415).

### Phylogenetic analysis

The COII alignment was translated into protein to examine the presence of stop codons using DnaSP software v5.10 [50]. Median-Joining haplotype networks were reconstructed for the guppy host and separately for each *Gyrodactylus* spp. using Network 4.611 software [51]. Maximum Likelihood and Bayesian Inference phylogenetic tree reconstructions were estimated for parasites using the software Garli 0.96 [52] and MrBayes v.2.1 [53], respectively. *G. salaris* and *G. thymalli* (GenBank acc. nos. NC008815 and NC009682, respectively) were used to root the *Gyrodactylus* spp. tree. Appropriate model of sequence evolution was chosen using jModeltest2 and the AIC criteria [54], and the GTR+G (-lnL = 1451) model was selected for phylogenetic tree reconstruction. Finally, to assess intraspecific and interspecific divergences, uncorrected *p*-distances were calculated within *Gyrodactylus* spp. and between each major clade using the software MEGA version 5 [55]. To assess typical levels of intraspecific diversity in *Gyrodactylus* spp., uncorrected *p*-distance was additionally calculated for the same 262 bp fragment for all published COII from other *Gyrodactylus* spp. available on GenBank (acc. nos. GU131204; GU131200; GU131198; GU131220, GU131214; GU131210; EU293891; NC009682 and NC008815). Substitution saturation was tested and rejected for the three current *Gyrodactylu*s spp. datasets (results not shown) using a test by Xia *et al*. [56, 57] implemented in software DAMBE 5.2.78 [58]. For guppies, uncorrected *p*-distances were calculated using the option to treat gaps as pairwise deletions.

### Population structure analysis

Haplotype and nucleotide diversities were calculated for the guppy and main phylogenetic lineages of *Gyrodactylus* spp. for each sampling locality whenever n ≥ 3 in DnaSP software v5.10 [50]. *F_ST_* measures were calculated for guppy and widespread lineages of *Gyrodactylus* spp. (see Results section) for each sampling locality whenever n ≥ 3 using Arlequin v3.5.1.3 [59].

### Assessment of host specificity levels

To test for any correlation between genetic differentiation of host and parasite populations, *F_ST_* values for each of the selected lineages of *Gyrodactylus* spp. were plotted against those of the guppy host and Pearson correlation coefficient was calculated for each parasite-host dyad in Minitab ver. 12.1.

### Morphometric analysis of *Gyrodactylus* specimens

Following results from the phylogenetic analyses, 25 individual point-to-point morphometric characteristics of the hamuli, ventral bar and marginal hooks (as outlined by [60]) were measured from those specimens clearly identified within a particular genetic lineage, and for which the posterior half of the body was of suitable quality. The aim of the morphometric analysis was to test whether these taxa were truly morphologically cryptic. To visualise multivariate patterns of morphological variation, a Principal Component Analysis (PCA) was conducted. To test whether genetic lineages where morphologically differentiated, a multivariate ANOVA based on permutations of the Euclidean Distance matrix between lineages mean values was performed. Post-hoc pairwise comparisons were also used to test whether there were significant morphological differences between cryptic lineages of *G. bullatarudis* (Gb1 and Gb2), as samples sizes were too small to test the same for the different lineages of *G. turnbulli* and *G. poeciliae*. All analyses were performed using available statistical R packages.

## Results

### Morphological identification of *Gyrodactylus* specimens

Morphological analyses of 131 *Gyrodactylus* specimens allowed the identification of three species: *G. poeciliae*, *G. turnbulli* and *G. bullatarudis*. While the latter two species had been already reported to parasitize *P. reticulata*, this is the first record of *G. poeciliae* infecting this host species.

### Phylogenetic analysis and macroevolutionary patterns of host and parasite species

Phylogenetic analysis of the guppy shows high phylogeographic structure of populations (see Fig. 1A). Three haplogroups are evident in haplotype network, which include: 1) most individuals from Aripo and 23 individuals from Lower Lopinot River (Caroni drainage); 2) individuals from Marianne River; and 3) individuals from the Oropouche, the remaining individuals from Lopinot and one individual from the Aripo River. Divergence amongst guppy individuals varied between 0 and 4.4%.

**Figure 2:**
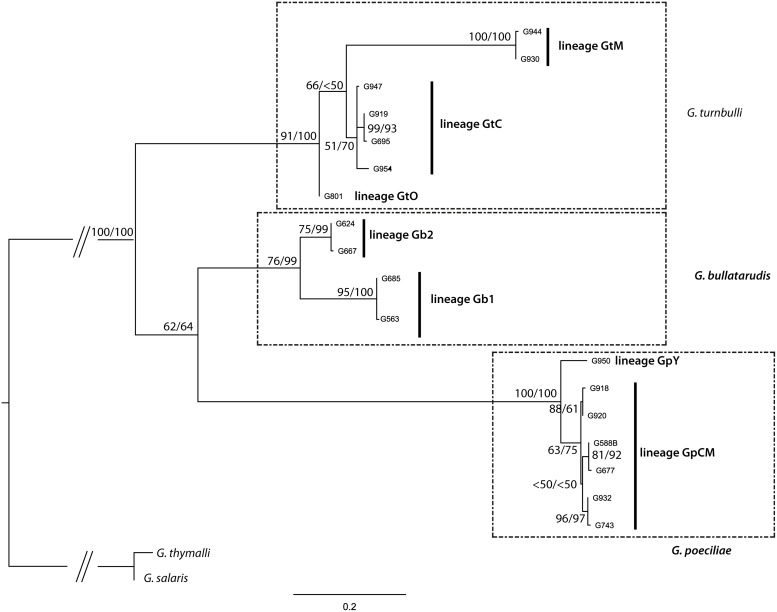
Best Maximum-likelihood tree obtained for the COII of the three *Gyrodactylus* study species. Values of nodes correspond to bootstrap support and Bayesian posterior probability, respectively. The three major clades corresponding to *Gyrodactylus* morphospecies are surrounded by a dotted line. Cryptic lineages within these clades are signaled with black bars.

Although our phylogenetic tree supports the monophyly of the three *Gyrodactylus* taxa (Fig. 2), divergence within *G. turnbulli*, *G. poeciliae* and *G. bullatarudis* varied between 0.4–17%, 0.4–6.9% and 0–13%, respectively. Since the values found for typical interspecific divergence of *Gyrodactylus* spp. range between 4% (*G. salaris vs G. thymalli*) and 39% (*e.g. G. thymalli vs G. corydori*), the intra-specific divergence found in the present study is equivalent to the typical interspecific divergence in all three cases, suggesting the presence of cryptic lineages.

For morphospecies *G. bullatarudis*, two phylogenetic lineages and haplogroups (Gb1 and Gb2) were detected (Figs. 1B and 2), although in this case, lineages/haplogroups overlapped in geographical range, both occurring in Aripo, Lopinot and Oropouche Rivers. Nonetheless, the two cryptic phylogenetic lineages from *G. bullatarudis* were retrieved as two very well supported sister clades (Fig. 2). Divergence within these lineages was low, ranging from 0–0.4%, and between lineages was reasonably high (12.6–13.0%).

For morphospecies *G. turnbulli*, three geographically segregated phylogenetic lineages and haplogroups were identified by both phylogenetic analyses (Figs. 1C and 2) distinguishing individuals from the Marianne River (GtM); Caroni drainage (GtC); and, an individual from the Oropouche River (GtO). Divergence within lineages varied between 0.4–3.4%, and between lineages was 5.7–7.3% between GtC *vs* GtO, 15.7–16.9% between GtC *vs* GtM and 16.9–17.2% between GtM *vs* GtO.

For morphospecies *G. poeciliae*, two apparently allopatric phylogenetic lineages and haplogroups were recovered by both phylogenetic analyses (Figs. 1D and 2), which distinguished two identical individuals collected from upper Yarra River (GpY) and the remaining individuals from the Caroni drainage and Marianne (GpCM). Divergence within GpCM varied between 0.4–2.3%, and divergence between the two lineages was 6.1–6.9%.

### Population structure and microevolutionary analysis of host-parasite coevolution

**Figure 3:**
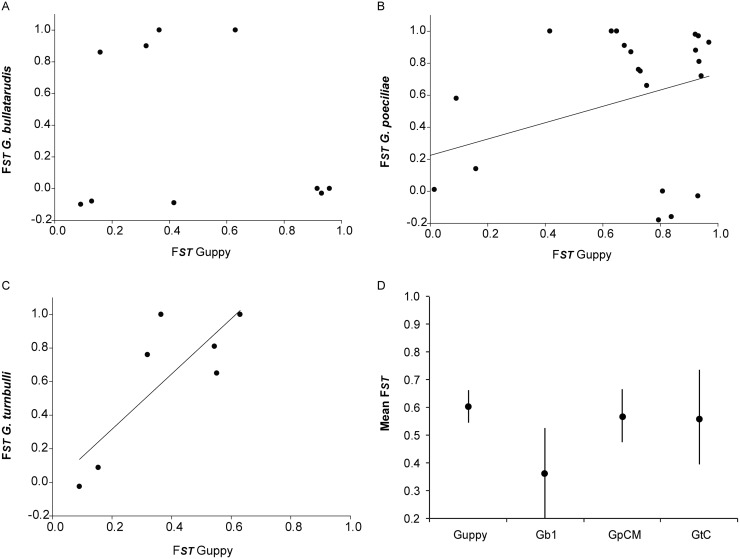
*F_ST_* plots for guppy host vs parasites of (A) lineage Gb1 of *Gyrodactylus bullatarudis*, (B) lineage GpCM of *G. poeciliae*, and (C) lineage GtC of *G. turnbulli*. Pearson’s correlation depicted if apparent. Additionally, mean *F_ST_* and SEM (standard error of the mean) are depicted in (D) for the guppy host and all 3 parasites.

Haplotype and nucleotide diversity for each *Gyrodactylus* lineage and guppy are depicted in Table 1. Haplotype diversity of morphospecies *G. bullatarudis* was generally low compared with the other two morphospecies. For the guppy, *F_st_* values showed high genetic differentiation between populations from different rivers (S1 Table). Comparing the pairwise *F_ST_* values of parasite populations (S1 Table) to the pairwise *F_ST_* of host populations showed that the level of genetic differentiation was approximately similar for host and parasites (all pairwise *t*-tests: *t*<1.75 *p*≥0.141). Interestingly, there was a significant positive correlation between *F_ST_* values of *G. poeciliae* GpCM and guppies (Pearson correlation coefficient *r* = 0.479, *p* = 0.027), and a marginally significant correlation (Pearson correlation coefficient *r* = 0.775, *p* = 0.070) between *F_ST_* values of GtC and guppies (see Fig. 3), but not for Gb1 (Pearson correlation coefficient *r* = -0.230, *p* = 0.522).

### Morphometric analysis of *Gyrodactylus* specimens

**Figure 4:**
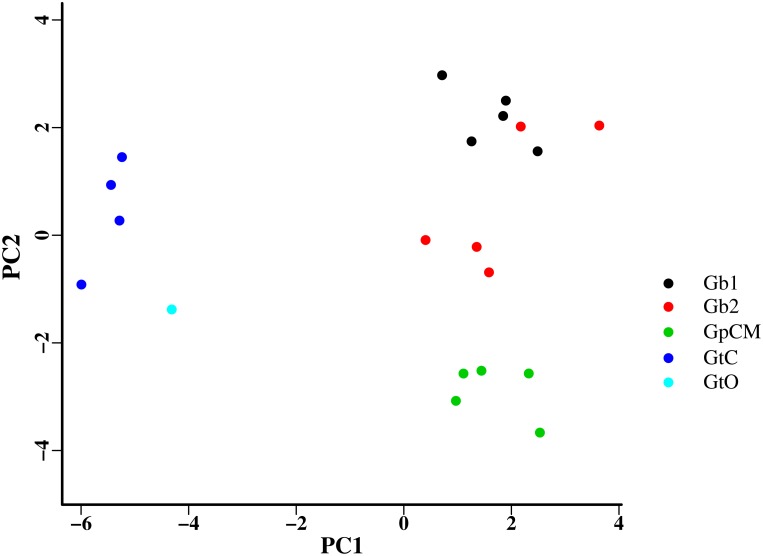
PCA plot showing morphological variation along PC1 and PC2 axes for *Gyrodactylus* lineages GtC, GtO, GpCM and Gb1 and Gb2.

Based on 25 measurements from the attachment hooks of each specimen (see S2 Table), PCA results highlighted high levels of morphological variation between *Gyrodactylus* lineages (GpCM, GtC, GtO, Gb1 and Gb2) examined, and PC1 and PC2 explained 42% and 18%, respectively, of the morphological variation found (Fig. 4) with PC3 and PC4 combined only contributing a further 16%. Differences between *G. turnbulli* (GtO and GtC) and the remaining *Gyrodactylus* species were clearly captured by PC1, whereas variation between GpCM, Gb1 and Gb2 were more evident along the PC2 axis, supporting the previous findings of Harris & Cable [21]. There was significant morphological differentiation between genetic lineages (ANOVA, F_4,15_ = 6.26, *p*<0.001).

## Discussion

Despite several studies having previously examined the genetic variability and structure of the guppy (*Poecilia reticulata*), the present dataset is the most comprehensive mitochondrial dataset of guppies in Trinidad. Our results confirm previous mtDNA studies and show that guppy population connectivity between different drainages is limited [38, 39]. There is evidence of migration from the Oropouche into the Caroni drainage, which has also been reported in recent SNP and microsatellite studies [40, 41]. Within rivers, gene flow appears to be in the downstream direction, with the lowlands populations of the Caroni drainage acting as a sink for immigrants from the upland habitats, which is consistent with previous studies [41, 43].

Regarding the guppy parasites, the three *Gyrodactylus* species examined each possess two to three genetic lineages, diverging by 5.7 to 17.2%. This is in the range of the divergence observed between published sequences for described species in this genus (4% divergence between *G. salaris vs G. thymalli*, and 39% between *G. thymalli vs G. corydori* [61–63]), which confirms that cryptic speciation is common within this genus [17]. In the case of *G. bullatarudis*, the two cryptic lineages had overlapping geographical ranges in both the Caroni and Oropouche drainages. Furthermore, for these two genetic lineages of *G. bullatarudis* there were no significant morphometric differences, confirming their cryptic nature. For *G. turnbulli*, the three cryptic lineages appeared to be geographically isolated in the Caroni, Oropouche and Marianne drainages. Within *G. poeciliae*, one lineage was found in the Yarra River (Northern drainage), and the other in both the Marianne River (Northern drainage) and Caroni drainage. However, while the divergence between lineages is evident in the haplotype network with both lineages being separated by a large number of mutations, phylogenetic support was low. Furthermore, as the *G. poeciliae* from the Yarra River were not included in the morphological analysis, the support for a cryptic species is much more tentative in *G. poeciliae* than for both other parasites. Remarkably, both for *G. poeciliae* and *G. turnbulli*, the level of genetic differentiation (expressed as pairwise population *F_ST_*) within lineages was comparable to that of the guppy host. Cryptic speciation and genetic diversification of the parasites may have occurred due to a variety of mechanisms including historical geographic isolation, co-evolution with multiple hosts and host switching, as will be discussed in the following section.

### Macroevolution in *Gyrodactylus* spp. resulted in cryptic variation

Poulin and Morand [64] postulated that the main reason for the high level of diversification among monogenean parasites is their small body size, which confers the ability for adaptation to different host micro-habitats, leading to sympatric speciation on a host. Such sympatric speciation has indeed been confirmed in *Dactylogyrus* gill parasites [65]. In the current study, however we assessed the genetic variation of viviparous rather than oviparous monogeneans [18]. *Gyrodactylus* spp. are renowned for their “Russian-Doll” reproductive strategy with one embryo developing inside another within the mother’s uterus [16]. Unlike *Dactylogyrus* and other oviparous monogeneans, they lack a free-living dispersal stage and the parasite infrapopulation can grow exponentially on a host with transmission occurring during host-host contact [16]. Cryptic lineages were not found to be co-infecting the same fish, and in the case of *G. turnbulli* and *G. poeciliae*, lineages did not even overlap geographically, although both *G. bullatarudis* lineages were found in the same host populations (but never on the same individual). The current results therefore do not support the hypothesis that intra host niche partitioning has resulted in sympatric speciation of *Gyrodactylus* spp. on the host.

The data from *G. turnbulli* and *G. poeciliae* seem to be most consistent with allopatric speciation, given that their lineages appeared to be geographically isolated. However, as *G. poeciliae* was originally described from *Poecilia caucana* [21], and the current study is the first to describe this parasite species on the guppy (*P. reticulata*), it is possible that Trinidadian populations of *G. poeciliae* may have also coevolved (and hence differentiated) on multiple host species.

The two cryptic lineages of *G. bullatarudis* had overlapping geographical ranges in both the Caroni and Oropouche drainages. This morphospecies is known to infect multiple hosts under laboratory conditions [34], and in Trinidad at least one lineage of *G. bullararudis* is able to infect killifish (*Anablepsoides hartii* [syn. = *Rivulus hartii*]) [36]. Individuals of *G. bullatarudis* can survive on killifish out of water for over an hour [36], which may confer a dispersal advantage as these fish can migrate overland. Unlike the guppy, gene flow between *G. bullatarudis* populations is not restricted by natural barriers in rivers, such as waterfalls and weirs [66], because the parasite could (theoretically) migrate overland when infecting a killifish. Hence, although the two cryptic lineages could have arisen due to allopatric isolation on a single host, it is also possible that divergence arose from co-evolution with different hosts. Secondary contact of the two lineages may have been easily facilitated by infection of other fish, such as the killifish, which would explain their current geographic overlap. Whichever was the case, both hypotheses are consistent with our observation that unlike the other parasite species, there was no positive correlation between all pairwise population *F_ST_* values of guppies with those of the *G. bullatarudis* populations.

### Conclusions

The current study represents the first evidence of cryptic genetic differentiation within Trinidadian *Gyrodactylus* species. We hypothesize that allopatric isolation and/or co-evolution with different hosts accounts for the extant species complexes. However, we fully acknowledge that such interpretations are only tentative at this stage because of the small sample sizes. Additionally, reported levels of differentiation should be confirmed with nuclear data to exclude the possibility of introgressive hybridisation [67]. The presence of multiple cryptic species could explain the difficulties previously encountered during development of microsatellite libraries for *Gyrodactylus* species of guppies [68]. The current findings will have important implications for future research using guppy-*Gyrodactylus* spp. dyads as models to test the impacts of parasites on host evolution [22, 69] and conservation planning [22, 31].

## Supporting Information

S1 Table Pairwise *F_ST_* for (A) guppies and (B) lineages of *Gyrodactylus poeciliae* (GpCM), *G. bullatarudis* (Gb1 and Gb2), and *G. turnbulli* (GtC), respectively. The symbol † corresponds to the only pairwise *F_ST_* estimated for lineage Gb2. Non-significant *F_ST_* values are highlighted in bold.
(DOCX)

S2 Table Morphological measurements of *Gyrodactylus bullatarudis*, *G. poeciliae* and *G. turnbulli* (mean±1 standard deviation followed by the range in parentheses). Measurements are provided in micrometres. Abbreviations: H = hamulus; VB = ventral bar; MH = marginal hook.
(DOCX)

## References

[1] Hafner MS, Page RDM (1995) Molecular phylogenies and host-parasite cospeciation: Gophers and lice as a model system. Philos T Roy Soc B 349: 77–83. 10.1098/rstb.1995.0093 8748020

[2] Hughes J, Kennedy M, Johnson KP, Palma RL, Page RD (2007) Multiple cophylogenetic analyses reveal frequent cospeciation between pelecaniform birds and *Pectinopygus* lice. Syst Biol 56: 232–251. 10.1080/10635150701311370 17464880

[3] Hoberg EP, Brooks DR (2008) A macroevolutionary mosaic: episodic host-switching, geographical colonization and diversification in complex host–parasite systems. J Biogeo 35: 1533–1550. 10.1111/j.1365-2699.2008.01951.x

[4] Peterson KR, Pfister DH (2010) Cophylogeny and biogeography of the fungal parasite *Cyttaria* and its host *Nothofagus*, southern beech. Mycologia 102: 1417–1425. 10.3852/10-048 20943538

[5] Jorge F, Roca V, Perera A, Harris DJ, Carretero MA (2011) A phylogenetic assessment of the colonisation patterns in *Spauligodon atlanticus* Astasio-Arbiza et al., 1987 (Nematoda: Oxyurida: Pharyngodonidae), a parasite of lizards of the genus *Gallotia* Boulenger: No simple answers. Syst Parasitol 80: 53–66. 10.1007/s11230-011-9311-1 21805391

[6] Dowton M, Austin AD (1995) Increased genetic diversity in mitochondrial genes is correlated with the evolution of parasitism in the Hymenoptera. J Mol Evol 41: 958–965. 10.1007/BF00173176 8587141

[7] Page RDM, Lee PLM, Becher SA, Griffiths R, Clayton DH (1998) A Different Tempo of Mitochondrial DNA Evolution in Birds and Their Parasitic Lice. Mol Phyl Evol 9: 276–293. 10.1006/mpev.1997.0458 9562986

[8] Light JE, Hafner MS (2007) Cophylogeny and disparate rates of evolution in sympatric lineages of chewing lice on pocket gophers. Mol Phyl Evol 45: 997–1013. 10.1016/j.ympev.2007.09.001 17964189

[9] Wu C, Li W (1985) Evidence for higher rates of nucleotide substitution in rodents than in man. PNAS 82: 1741–1745. 10.1073/pnas.82.6.1741 3856856 PMC397348

[10] Huyse T, Poulin R, Theron A (2005) Speciation in parasites: a population genetics approach. Trends Parasitol 21: 469–475. 10.1016/j.pt.2005.08.009 16112615

[11] Barson M, Přikrylová I, Vanhove MPM, Huyse T (2010) Parasite hybridization in African *Macrogyrodactylus* spp. (Monogenea, Platyhelminthes) signals historical host distribution. Parasitology 137: 1585–1595. 10.1017/S0031182010000302 20444301

[12] Pariselle A, Boeger WA, Snoeks J, Bilong CFB, Morand S, et al (2011) The monogenean parasite fauna of cichlids: a potential tool for host biogeography. Int J Evol Biol 2011: 471–480. 10.4061/2011/471480 21869935 PMC3157826

[13] Bakke TA, Jansen PA, Hansen LP (1990) Differences in the host resistance of Atlantic salmon (*Salmo salar*) stocks to the monogenean *Gyrodactylus salaris* Malmberg, 1957. J Fish Biol 37: 577–587. 10.1111/j.1095-8649.1990.tb05890.x

[14] Cable J, Harris PD, Tinsley RC (1996) Ultrastructural adaptations for viviparity in the female reproductive system of gyrodactylid monogeneans. Tissue Cell 28: 515–526. 10.1016/S0040-8166(96)80054-1 18621336

[15] Cable J, Harris PD, Tinsley RC, Lazarus CM (1999) Phylogenetic analysis of *Gyrodactylus* spp. (Platyhelminthes: Monogenea) using ribosomal DNA sequences. Can J Zool 77: 1439–1449.

[16] Bakke TA, Cable J, Harris PD (2007) The biology of gyrodactylid monogeneans: the “Russian-Doll killers”. Adv Parasit 64: 459–460. 17499102 10.1016/S0065-308X(06)64003-7

[17] Hansen H, Bakke TA, Bachmann L (2007) DNA taxonomy and barcoding of monogenean parasites: lessons from *Gyrodactylus* . Trends Parasitol 23: 363–367. 10.1016/j.pt.2007.06.007 17602869

[18] Cable J, Harris PD, Tinsley RC (1998) Life history specializations of monogenean flatworms: a review of experimental and microscopical studies. Microsc Res Techniq 42: 186–199. 10.1002/(SICI)1097-0029(19980801)42:3<186::AID-JEMT3>3.3.CO;2-J 9764919

[19] Harris PD, Shinn AP, Cable J, Bakke TA, Bron J (2008) GyroDb: gyrodactylid monogeneans on the web. Trends Parasitol 24: 109–111. 10.1016/j.pt.2007.12.004 18243795

[20] Huyse T, Volckaert FAM (2002) Identification of a host-associated species complex using molecular and morphometric analyses, with the description of *Gyrodactylus rugiensoides* n. sp. (Gyrodactylidae, Monogenea). Int J Parasitol 32: 907–919. 10.1016/S0020-7519(02)00026-7 12062562

[21] Harris PD, Cable J (2000). *Gyrodactylus poeciliae* n. sp. and *G. milleri* n. sp. (Monogenea: Gyrodactylidae) from *Poecilia caucana* (Steindachner) in Venezuela. Syst Parasitol 47:79–85. 10.1023/A:1006413804061 10966215

[22] Cable J, van Oosterhout C (2007) The impact of parasites on the life history evolution of guppies (*Poecilia reticulata*): The effects of host size on parasite virulence. Int J Parasitol 37: 1449–1458. 10.1016/j.ijpara.2007.04.013 17561023

[23] Kennedy CEJ, Endler JA, Poynton SL, McMinn H (1987) Parasite load predicts mate choice in guppies. Behav Ecol Sociobiol 21: 291–295. 10.1007/BF00299966

[24] Kolluru GR, Grether GF, Dunlop E, South SH (2008) Food availability and parasite infection influence mating tactics in guppies (*Poecilia reticulata*). Behav Ecol 20:131–137. 10.1093/beheco/arn124

[25] Jeswiet SB, Lee-Jenkins SSY, Ramnarine IW, Godin J-GJ (2011) Sperm competition risk and mate choice in male Trinidadian guppies, *Poecilia reticulata* . Anim Behav 81: 639–644. 10.1016/j.anbehav.2010.12.013

[26] Scott ME (1985) Dynamics of challenge infections of *Gyrodactylus bullatarudis* Turnbull (Monogenea) on guppies, *Poecilia reticulata* (Peters). J Fish Dis 8: 495–503. 10.1111/j.1365-2761.1985.tb00964.x

[27] van Oosterhout C, Joyce DA, Cummings SM, Blais J, Barson NJ, et al (2006) Balancing selection, random genetic drift, and genetic variation at the major histocompatibility complex in two wild populations of guppies (*Poecilia reticulata*). Evolution 60: 2562–2574. 10.1111/j.0014-3820.2006.tb01890.x 17263117

[28] Cable J, van Oosterhout C (2007) The role of innate and acquired resistance in two natural populations of guppies (*Poecilia reticulata*) infected with the ectoparasite *Gyrodactylus turnbulli* . Biol J Linn Soc 90: 647–655. 10.1111/j.1095-8312.2006.00755.x

[29] Richards EL, van Oosterhout C, Cable J (2010) Sex-specific differences in shoaling affect parasite transmission in guppies. PLoS ONE 5: e13285 10.1371/journal.pone.0013285 20949014 PMC2952601

[30] Johnson MB, Lafferty KD, van Oosterhout C, Cable J (2011) Parasite transmission in social interacting hosts: monogenean epidemics in guppies. PLoS ONE 6: e22634 10.1371/journal.pone.0022634 21897838 PMC3163578

[31] van Oosterhout C, Smith AM, Hänfling B, Ramnarine IW, Mohammed RS, et al (2007) The guppy as a conservation model: Implications of parasitism and inbreeding for reintroduction success. Conserv Biol 21: 1573–1583. 18173481 10.1111/j.1523-1739.2007.00809.x

[32] Faria P, van Oosterhout C, Cable J (2010) Optimal release strategies for captive-bred animals in reintroduction programs: the effects of prior parasite exposure and release protocol on host survival and infection rates. Biol Conserv 143: 35–41. 10.1016/j.biocon.2009.06.002

[33] Malmberg G (1970) The excretory systems and the marginal hooks as a basis for the systematics of *Gyrodactylus* (Trematoda, Monogenea). Arkiv for Zoologie 23:1–237.

[34] King TA, Cable J (2007) Experimental infections of the monogenean *Gyrodactylus turnbulli* indicate that it is not a strict specialist. Int J Parasitol 37: 663–672. 10.1016/j.ijpara.2006.11.015 17224155

[35] King TA, van Oosterhout C, Cable J (2009) Experimental infections with the tropical monogenean, *Gyrodactylus bullatarudis*: Potential invader or experimental fluke? Parasitol Int 58: 249–254. 10.1016/j.parint.2009.04.005 19394437

[36] Cable J, Archard GA, Mohammed RS, McMullan M, Stephenson JF, et al (2013) Can parasites use predators to spread between primary hosts? Parasitology 140: 1–6. 10.1017/S003118201300067X 23714691

[37] Cable J, van Oosterhout C, Barson NJ, Harris PD (2005) *Gyrodactylus pictae* n. sp. (Monogenea: Gyrodactylidae) from the Trinidadian swamp guppy *Poecilia picta* Regan, with a discussion on species of *Gyrodactylus* von Nordmann, 1832 and their poeciliid hosts. Syst Parasitol 60: 159–164. 10.1007/s11230-004-6348-4 15864453

[38] Shaw PW, Carvalho GR, Magurran AE, Seghers BH (1991) Population differentiation in Trinidadian guppies, *Poecilia reticulata*—patterns and problems. J Fish Biol 39: 203–209. 10.1111/j.1095-8649.1991.tb05084.x

[39] Fajen A, Breden F (1992) Mitochondrial DNA sequence variation among natural populations of the Trinidad gyppy, *Poecilia reticulata* . Evolution 46: 1457–1465. 10.2307/2409949 28568990

[40] Suk H, Neff B (2009) Microsatellite genetic differentiation among populations of the Trinidadian guppy. Heredity 102: 425–434. 10.1038/hdy.2009.7 19223925

[41] Willing E-A, Bentzen P, Van Oosterhout C, Hoffmann M, Cable J, et al (2010) Genome-wide single nucleotide polymorphisms reveal population history and adaptive divergence in wild guppies. Mol Ecol 19: 968–984. 10.1111/j.1365-294X.2010.04528.x 20149094

[42] Crispo E, Bentzen P, Reznick DN, Kinnison MT, Hendry AP (2006) The relative influence of natural selection and geography on gene flow in guppies. Mol Ecol 15: 49–62. 10.1111/j.1365-294X.2005.02764.x 16367829

[43] Barson NJ, Cable J, van Oosterhout C (2009) Population genetic analysis of microsatellite variation of guppies (*Poecilia reticulata*) in Trinidad and Tobago: evidence for a dynamic source-sink metapopulation structure, founder events and population bottlenecks. J Evol Biol 22: 485–497. 10.1111/j.1420-9101.2008.01675.x 19210594

[44] Stephenson JF, van Oosterhout C, Mohammed RS, Cable J (2014) Parasites of Trinidadian guppies: evidence for sex- and age-specific trait-mediated indirect effects of predators. Ecology: In press. 10.1890/14-0495.1 26240870

[45] Paladini G, Gustinelli A, Fioravanti ML, Hansen H, Shinn AP (2009) The first report of *Gyrodactylus salaris* Malmberg, 1957 (Platyhelminthes, Monogenea) on Italian cultured stocks of rainbow trout (*Oncorhynchus mykiss* Walbaum). Vet Parasitol 165: 290–297. 10.1016/j.vetpar.2009.07.025 19700245

[46] Harris PD, Cable J, Tinsley RC, Lazarus CM (1999) Combined ribosomal DNA and morphological analysis of individual gyrodactylid monogeneans. J Parasitol 85: 188–191. 10.2307/3285617 10219293

[47] Truett GE, Heeger P, Mynatt RL, Truett AA, Walker JA, et al (2000) Preparation of PCR-quality mouse genomic DNA with hot sodium hydroxide and Tris (HotSHOT). BioTechniques 29:52–54. 10907076 10.2144/00291bm09

[48] Kocher TD, Thomas WK, Meyer A, Edwards SV, Pääbo S, et al (1989) Dynamics of mitochondrial DNA evolution in animals: Amplification and sequencing with conserved primers. PNAS 86: 6196–6200. 10.1073/pnas.86.16.6196 2762322 PMC297804

[49] Shields GF, Kocher TD (1991) Phylogenetic relationships of north American ursids based on analysis of mitochondrial DNA. Evolution 45: 218–221. 10.2307/2409495 28564083

[50] Librado P, Rozas J (2009) DnaSP v5: A software for comprehensive analysis of DNA polymorphism data. Bioinformatics 25: 1451–1452. 10.1093/bioinformatics/btp187 19346325

[51] Bandelt H-J, Forster P, Röhl A (1999) Median-joining networks for inferring intraspecific phylogenies. Mol Biol Evol 16: 37–48. 10.1093/oxfordjournals.molbev.a026036 10331250

[52] Zwickl DJ (2006) Genetic algorithm approaches for the phylogenetic analysis of large biological sequence datasets under the maximum likelihood criterion. Ph.D. thesis: The University of Texas at Austin, USA.

[53] Ronquist F, Teslenko M, van der Mark P, Ayres D, Darling A, et al (2012) MrBayes 3.2: Eficient Bayesian phylogenetic inference and model choice across a large model space. Syst Biol 61:539–542. 10.1093/sysbio/sys029 22357727 PMC3329765

[54] Darriba D, Taboada GL, Doallo R, Posada D (2012). jModelTest 2: more models, new heuristics and parallel computing. Nature Methods 9:772 10.1038/nmeth.2109 22847109 PMC4594756

[55] Tamura K, Peterson D, Peterson N, Stecher G, Nei M, et al (2011) MEGA5: Molecular evolutionary genetics analysis using Maximum Likelihood, evolutionary distance, and Maximum Parsimony methods. Mol Biol Evol 28: 2731–2739. 10.1093/molbev/msr121 21546353 PMC3203626

[56] Xia X, Xie Z, Salemi M, Chen L, Wang Y (2003) An index of substitution saturation and its application. Mol Phyl Evol 26: 1–7. 10.1016/S1055-7903(02)00326-3 12470932

[57] Xia X, Lemey P (2009) Assessing substitution saturation with DAMBE. In: Lemey P, Salemi M, Vandamme A-M editors. The Phylogenetic Handbook: A Practical Approach to DNA and Protein Phylogeny. pp. 615–630. Cambridge University Press.

[58] Xia X, Xie Z (2001) DAMBE: Data analysis in molecular biology and evolution. J Hered 92: 371–373. 10.1093/jhered/92.4.371 11535656

[59] Excoffier L, Lischer HEL (2010) Arlequin suite ver 3.5: A new series of programs to perform population genetics analyses under Linux and Windows. Mol Ecol Resour 10: 564–567. 10.1111/j.1755-0998.2010.02847.x 21565059

[60] Shinn AP, Hansen H, Olstad K, Bachmann L, Bakke TA (2004) The use of morphometric characters to discriminate species of laboratory-reared and wild populations of *Gyrodactylus salaris* and *G. thymalli* (Monogenea). Folia Parasitol 51: 239–252. 10.14411/fp.2004.029 15357403

[61] Huyse T, Buchmann K, Littlewood DT (2008) The mitochondrial genome of *Gyrodactylus derjavinoides* (Platyhelminthes: Monogenea)- a mitogenomic approach for *Gyrodactylus* species and strains identification. Gene 417: 27–34. 10.1016/j.gene.2008.03.008 18448274

[62] Plaisance L, Huyse T, Littlewood DT, Bakke TA, Bachmann L (2007) The complete mitochondrial DNA sequence of the monogenean *Gyrodactylus thymalli* (Platyhelmithes: Monogenea), a parasite of grayling (*Thymallus thymallus*). Mol Biochem Parasitol 154: 190–194. 17559954 10.1016/j.molbiopara.2007.04.012

[63] Bueno-Silva M, Boeger W (2014) Neotropical Monogenoidea. 58. Three new species of *Gyrodactylus* (Gyrodactylidae) from *Scleromystax* spp. (Callichtyidae) and the proposal of COII gene as an additional fragment for barcoding gyrodactylids. Folia Parasitol 61: 213–222. 10.14411/fp.2014.028 25065127

[64] Poulin R, Morand S (2000). The diversity of parasites. Q Rev Biol 75: 277–293. 10.1086/393500 11008700

[65] Šimková A, Morand S, Jobet E, Gelnar M, Verneau O (2004) Molecular phylogeny of congeneric monogenean parasites (*Dactylogyrus*): a case of intrahost speciation. Evolution 58: 1001–1018. 10.1111/j.0014-3820.2004.tb00434.x 15212381

[66] Reznick DN (1995) Life history evolution in guppies: a model system for the empirical study of adaptation. Neth J Zool 46: 172–190. 10.1163/156854295X00140

[67] Ziȩtara MS, Rokicka M, Stojanovski S, Lumme J (2010) Introgression of distant mitochondria into the genome of *Gyrodactylus salaris*: Nuclear and mitochondrial markers are necessary to identify parasite strains. Acta Parasitol 55: 20–28. 10.2478/s11686-010-0016-4

[68] Faria PJ, Lazarus CM, van Oosterhout C, Harris PD, Cable J (2011) First polymorphic microsatellites for the gyrodactylids (Monogenea), an important group of fish pathogens. Conserv Genet Resour 3: 177–180. 10.1007/s12686-010-9317-z

[69] Pérez-Jvostov F, Hendry A, Fussmann G, Scott M (2012) Are host-parasite interactions influenced by adaptation to predators? A test with guppies and *Gyrodactylus* in experimental stream channels. Oecologia 170: 77–88. 10.1007/s00442-012-2289-9 22402622

