# Supplementary material for: Evidence for Cryptic Speciation in Directly Transmitted Gyrodactylid Parasites of Trinidadian Guppies
Source: PLoS One. 2015 Jan 9;10(1):e0117096. doi: 10.1371/journal.pone.0117096 (PMC4289073; doi:10.1371/journal.pone.0117096)
Supplement: S1 Table — The symbol † corresponds to the only pairwise FST estimated for lineage Gb2. Non-significant FST values are highlighted in bold. (DOCX) [file pone.0117096.s001.docx]

Table S1. Pairwise *F_ST_* for (A) guppies and (B) lineages of *Gyrodactylus poeciliae* (GpCM), *G. bullatarudis* (Gb1 and Gb2), and *G. turnbulli* (GtC), respectively. The symbol † corresponds to the only pairwise *F_ST_* estimated for lineage Gb2. Non-significant *F_ST_* values are highlighted in bold.

Table S1A: Pairwise *F_ST_* for guppies.

|  | Marianne | U Marianne | L Marianne | Aripo | U Aripo | L Aripo | Lopinot | U Lopinot | L Lopinot |
| --- | --- | --- | --- | --- | --- | --- | --- | --- | --- |
| Marianne |  |  |  |  |  |  |  |  |  |
| U Marianne | **-0.01244** |  |  |  |  |  |  |  |  |
| L Marianne | **-0.01296** | **0.01479** |  |  |  |  |  |  |  |
| Aripo | 0.73067 | 0.72425 | 0.75288 |  |  |  |  |  |  |
| U Aripo | 0.79407 | 0.80799 | 0.83857 | 0.09094 |  |  |  |  |  |
| L Aripo | 0.67507 | 0.64838 | 0.69813 | 0.12941 | 0.41612 |  |  |  |  |
| Lopinot | na | na | na | 0.55185 | 0.54408 |  |  |  |  |
| U Lopinot | 0.92315 | 0.92172 | 0.93488 | 0.94244 | 0.96903 | 0.93257 | na |  |  |
| L Lopinot | 0.46229 | na | 0.41421 | 0.31927 | 0.36462 | 0.15905 | 0.15418 | na |  |
| L Oropouche | na | na | na | 0.93084 | 0.95834 | 0.91559 | na | na | 0.63012 |
|  |  |  |  |  |  |  |  |  |  |

Table S1B- Pairwise *F_ST_* obtained for lineages of *Gyrodactylus poeciliae* (GpCM), *G. bullatarudis* (Gb1 and Gb2), and *G. turnbulli* (GtC), respectively.

|  | Marianne | U Marianne | L Marianne | Aripo | U Aripo | L Aripo | Lopinot | U Lopinot | L Lopinot |
| --- | --- | --- | --- | --- | --- | --- | --- | --- | --- |
| Marianne |  |  |  |  |  |  |  |  |  |
| U Marianne | **0.01**\na\na |  |  |  |  |  |  |  |  |
| L Marianne | **-0.02**\na\na | **0.01** |  |  |  |  |  |  |  |
| Aripo | 0.75\na\na | 0.76 | 0.66\na\na |  |  |  |  |  |  |
| U Aripo | **-0.18**\na\na | **0** | **-0.16**\na\na | 0.58\-**0.1**\-0.025 |  |  |  |  |  |
| L Aripo | 0.91\na\na | 1 | 0.87\na\na | na\-**0.08**\na | 1\-**0.09**\na |  |  |  |  |
| Lopinot | na | na | na | na\na\0.65 | na\na\0.81 | na |  |  |  |
| U Lopinot | 0.88\na\na | 0.98\na\na | 0.81\na\na | 0.72\na\na | 0.93\na\na | 0.97\na\na | na |  |  |
| L Lopinot | na | na | na | na\0.9\0.76 | na\1\1 | 0.14\0.86\na | na\na\**0.09** | na |  |
| L Oropouche | na | na | na | na**\-0.03**\na | na\**0**.**0**†\na | na\**0**\na | na | na | na\1\na |
