# Supplementary material for: Evidence for Cryptic Speciation in Directly Transmitted Gyrodactylid Parasites of Trinidadian Guppies
Source: PLoS One. 2015 Jan 9;10(1):e0117096. doi: 10.1371/journal.pone.0117096 (PMC4289073; doi:10.1371/journal.pone.0117096)
Supplement: S2 Table — Measurements are provided in micrometres. Abbreviations: H = hamulus; VB = ventral bar; MH = marginal hook. (DOCX) [file pone.0117096.s002.docx]

Table S2. Morphological measurements of *Gyrodactylus bullatarudis*, *Gyrodactylus poeciliae* and *Gyrodactylus turnbulli* (mean±1 standard deviation followed by the range in parentheses). Measurements are provided in micrometres. Abbreviations: H = hamulus; VB = ventral bar; MH = marginal hook.

| **Variable** | ***Gyrodactylus bullatarudis*** | | ***Gyrodactylus poeciliae*** | ***Gyrodactylus turnbulli*** | |
| --- | --- | --- | --- | --- | --- |
|  | **Gb1**  **(n = 5)** | **Gb2**  **(n = 5)** | **GpCM**  **(n = 5)** | **GtC**  **(n = 5)** | **GtO**  **(n = 1)** |
| H aperture | 18.1±1.3 (16.0–19.8) | 17.3±1.4 (15.8–19.1) | 18.1±0.4 (17.6–18.5) | 23.5±0.9 (22.2–24.1) | 21.46 |
| H proximal shaft width | 10.2±1.6 (8.4–12.3) | 10.1±0.3 (9.9–10.6) | 9.4±0.2 (9.2–9.7) | 10.6±0.2 (10.3–10.9) | 10.11 |
| H point length | 27.0±0.9 (25.8–28.1) | 25.7±1.8 (22.8–27.3) | 23.1±0.8 (22.3–24.1) | 26.7±0.6 (25.9–27.2) | 25.57 |
| H distal shaft width | 5.0±0.8 (4.4–6.3) | 5.0±0.7 (4.2–5.9) | 4.6±0.3 (4.2–5.0) | 4.4±0.6 (4.0–5.4) | 4.62 |
| H shaft length | 38.3±1.2 (36.7–39.6) | 37.0±0.9 (36.1–38.1) | 35.2±0.6 (34.7–35.9) | 40.9±0.7 (40.1–41.7) | 37.98 |
| H inner curve length | 1.8±0.6 (1.4–2.9) | 2.4±0.6 (1.8–3.1) | 3.5±0.2 (3.2–3.7) | 3.4±0.5 (2.7–3.9) | 4.24 |
| H aperture angle (°) | 33.9±2.1 (31.8–37.1) | 33.9±3.7 (30.1–37.9) | 37.3±1.2 (36.4–39.2) | 41.4±1.0 (40.1–42.2) | 41.41 |
| H point curve angle (°) | 5.1±1.4 (4.1–7.6) | 7.6±2.7 (4.8–10.6) | 12.5±1.5 (10.8–14.4) | 11.5±2.1 (8.3–12.9) | 14.77 |
| Inner aperture angle (°) | 39.3±2.8 (37.8–44.3) | 39.2±4.3 (35.0–44.9) | 43.0±1.3 (41.5–44.4) | 45.2±1.3 (43.6–46.6) | 47.52 |
| H root length | 14.6±1.6 (12.4–16.5) | 16.5±1.1 (15.1–18.1) | 16.5±0.8 (15.4–17.5) | 19.1±0.6 (18.4–19.8) | 17.29 |
| H total length | 53.2±1.5 (51.2–54.7) | 53.9±0.5 (53.2–54.4) | 52.1±0.3 (51.6–52.5) | 59.2±1.7 (57.1–61.3) | 57.21 |
| VB total width | 33.6±1.6 (32.2–36.1) | 30.3±0.7 (29.7–31.4) | 29.7±1.0 (28.3–30.9) | 31.5±5.7 (26.1–39.4) | 35.11 |
| VB total length | 28.8±0.4 (28.4–29.1) | 29.5±2.3 (26.1–32.0) | 26.2±0.9 (25.1–27.3) | 30.3±1.6 (28.4–32.3) | 29.77 |
| VB process-to-mid length | 7.7±1.1 (6.4–8.7) | 7.3±0.3 (6.8–7.5) | 6.5±1.0 (5.4–8.1) | 5.4±1.2 (4.3–6.7) | 6.38 |
| VB median length | 6.8±0.9 (5.5–7.7) | 7.0±1.0 (5.9–8.3) | 6.5±0.6 (5.9–7.2) | 8.9±0.1 (8.8–9.0) | 6.81 |
| VB process length | 7.5±0.5 (7.0–8.2) | 7.2±0.5 (6.5–7.9) | 6.5±0.4 (5.9–7.0) | 7.5±0.7 (6.5–8.3) | 5.42 |
| VB membrane length | 14.5±1.0 (13.1–15.6) | 15.4±1.8 (13.2–17.8) | 13.6±0.6 (12.9–14.6) | 14.1±6.8 (3.9–17.9) | 17.16 |
| MH total length | 25.6±1.1 (24.7–27.5) | 24.2±1.5 (21.6–25.3) | 23.6±0.5 (23.1–24.5) | 32.7±0.7 (31.7–33.5) | 34.4 |
| MH shaft length | 20.5±0.6 (20.2–21.6) | 19.3±1.5 (16.8–20.7) | 18.1±0.8 (17.4–19.3) | 25.5±0.8 (24.4–26.2) | 26.64 |
| MH sickle length | 5.5±0.1 (5.3–5.6) | 5.4±0.2 (5.2–5.7) | 5.7±0.4 (5.3–6.1) | 7.8±0.1 (7.7–7.9) | 7.7 |
| MH sickle proximal width | 4.1±0.4 (3.5–4.6) | 3.8±0.3 (3.5–4.3) | 4.0±0.2 (3.9–4.3) | 4.2±0.1 (4.1–4.3) | 4.5 |
| MH toe length | 2.0±0.2 (1.8–2.4) | 1.8±0.2 (1.4–2.0) | 2.0±0.1 (1.8–2.1) | 2.0±0.1 (1.9–2.0) | 1.82 |
| MH sickle distal width | 1.9±0.1 (1.8–2.1) | 1.7±0.2 (1.5–1.9) | 1.5±0.2 (1.3–1.7) | 4.5±0.3 (4.5–4.8) | 4.32 |
| MH aperture | 5.2±0.2 (5.0–5.5) | 5.1±0.1 (5.0–5.2) | 5.3±0.5 (4.8–6.0) | 7.1±0.2 (6.9–7.3) | 6.86 |
| MH instep / arch height | 0.5±0.2 (0.3–0.7) | 0.5±0.1 (0.4–0.7) | 0.6±0.1 (0.5–0.6) | 0.4±0.1 (0.3–0.5) | 0.38 |
